# Using Low Temperature Photoluminescence Spectroscopy to Investigate CH_3_NH_3_PbI_3_ Hybrid Perovskite Degradation

**DOI:** 10.3390/molecules21070885

**Published:** 2016-07-08

**Authors:** Khaoula Jemli, Hiba Diab, Ferdinand Lédée, Gaelle Trippé-Allard, Damien Garrot, Bernard Geffroy, Jean-Sébastien Lauret, Pierre Audebert, Emmanuelle Deleporte

**Affiliations:** 1 Laboratoire Aimé Cotton, Ecole Normale Supérieure de Cachan, CNRS, Université Paris-Sud, Université Paris-Saclay, Bât 505 Campus d’Orsay, 91405 Orsay, France; kjemli@ens-cachan.fr (K.J.); hiba.diab@ens-cachan.fr (H.D.); ferdinand.ledee@ens-cachan.fr (F.L.); gaelle.allard@ens-cachan.fr (G.T.-A.); jean-sebastien.lauret@lac.u-psud.fr (J.-S.L.); 2 Laboratoire de Photophysique et Photochimie Supramoléculaires et Macromoléculaires de l’Ecole Normale Supérieure de Cachan, 61 Avenue du Président Wilson, 94235 Cachan, France; 3 Groupe d’Etudes de la Matière Condensée (GEMaC), CNRS, Université de Versailles Saint-Quentin-en-Yvelines, Université Paris-Saclay, 45 Avenue des Etats-Unis, 78035 Versailles Cedex, France; damien.garrot@uvsq.fr; 4 Laboratory of Innovation in Surface Chemistry and Nanosciences, NIMBE, CEA, CNRS, Université Paris-Saclay, CEA Saclay, 91191 Gif-sur-Yvette Cedex, France; bernard.geffroy@polytechnique.edu

**Keywords:** CH_3_NH_3_PbI_3_, hybrid perovskite, solar cells, optoelectronics, degradation, photoluminescence

## Abstract

Investigating the stability and evaluating the quality of the CH_3_NH_3_PbI_3_ perovskite structures is quite critical both to the design and fabrication of high-performance perovskite devices and to fundamental studies of the photophysics of the excitons. In particular, it is known that, under ambient conditions, CH_3_NH_3_PbI_3_ degrades producing some PbI_2_. We show here that low temperature Photoluminescence (PL) spectroscopy is a powerful tool to detect PbI_2_ traces in hybrid perovskite layers and single crystals. Because PL spectroscopy is a signal detection method on a black background, small PbI_2_ traces can be detected, when other methods currently used at room temperature fail. Our study highlights the extremely high stability of the single crystals compared to the thin layers and defects and grain boundaries are thought to play an important role in the degradation mechanism.

## 1. Introduction

The easy synthesis by low-cost technologies of layered hybrid organic-inorganic perovskites appeared has been known for less than 30 years, and it has become more and more attractive in the optoelectronics field [1,2,3,4,5,6,7,8,9,10]. A scientific breakthrough was achieved in the photovoltaic domain in 2009 with the 3D hybrid perovskite CH_3_NH_3_PbI_3_, thanks to several remarkable properties such as ambipolar transport properties, electron-hole diffusion lengths exceeding 1 micrometer [11,12], high mobilities, large spectral absorption from the near-infrared range [13], and efficient charge separation [14,15]. Huge and rapid progress has been made in a short period of time since 2012 [16,17,18,19,20,21,22,23,24,25,26,27], leading to the recent record of 22.1% for the power conversion efficiency [28]. Despite this runaway success, some critical points remain, such as the environmental problem of the lead use [29,30], the poor photostability [31] and the sensitivity to atmospheric moisture [32,33,34,35,36,37,38,39,40]. These points have absolutely to be addressed to promote the use of hybrid perovskites in optoelectronic devices, such as solar cells, at a large scale.

It is now established that the problem of the aging of the solar cells is largely due to the aging of the CH_3_NH_3_PbI_3_ (called MAPI hereafter) layer itself [21]. The degradation of the MAPI perovskite due to moisture has been particularly studied recently [32,36,37,38,39,40]. Niu and coworkers have proposed a mechanism for the degradation of MAPI, in which the final products of the degradation are PbI_2_ and I_2_ [36].

**Figure 1:**
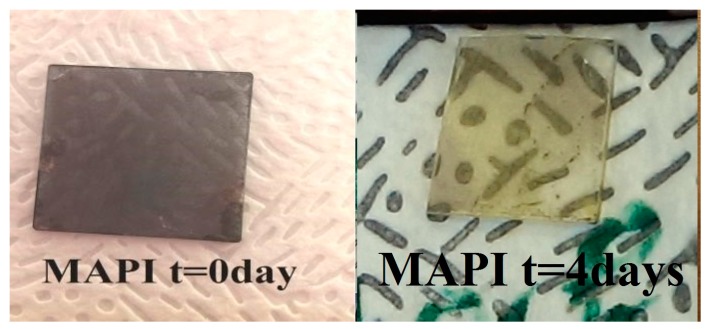
Photography of a 800 nm thick CH_3_NH_3_PbI_3_ layer deposited by spin-coating on a quartz substrate, just after the deposition (t = 0 day) and 4 days after the deposition (t = 4 days).

**Figure 2:**
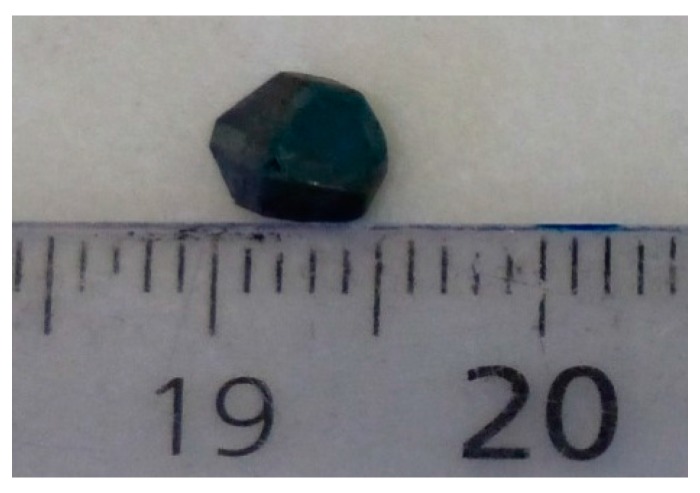
Photography of a millimeter sized MAPI crystal, 6 months after its growth.

Figure 1 shows a photograph of a 800 nm-thick MAPI layer deposited by spin-coating on a quartz substrate. Just after the deposition, the sample appears grey, but only 4 days later it appears completely yellow due to the apparition of PbI_2_ inside the layer [34]. On the contrary, Figure 2 shows the photography of a millimeter sized MAPI crystal aged six months, and no trace of PbI_2_ can be detected by the naked eye. Nevertheless, it is very important to check if traces of PbI_2_ exist in this crystal or not, even if these traces are very small, because it is usually believed that hydrates are harmful to the transport properties, as insulating layers are probably formed at the frontier between grain boundaries [38].

Characterization means currently used to detect the presence of PbI_2_ are absorption spectroscopy and X-diffraction analysis performed at room temperature [31,32,34,35,36,37]. Recently, other methods have been proposed: ellipsometry [38] and in situ electrical resistance measurement [39]. We propose here the use of photoluminescence (PL) spectroscopy at low temperature as a powerful tool to detect the presence of PbI_2_ traces and to finely characterize the aging of MAPI layers and crystals using this technique. In the particular case of MAPI crystals, X-diffraction and absorption spectroscopies are inadequate to detect small PbI_2_ traces, so the only way to efficiently manage to this goal, without destroying or changing the nature of the sample, is to use low temperature PL spectroscopy, which is an in situ characterization method.

## 2. Results and Discussion

### 2.1. Optical Properties of Thin Layers

**Figure 3:**
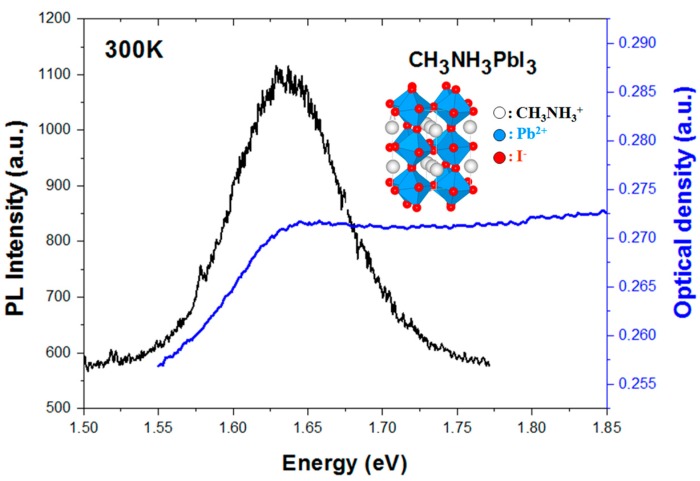
Absorption spectrum (blue curve) and photoluminescence spectrum (black curve) of a 800 nm thick MAPI layer at room temperature, taken just after the deposition by spin-coating (t = 0 day). The excitation wavelength is 3.815 eV (325 nm, HeCd laser). The inset exhibits the molecular structure of CH_3_NH_3_PbI_3_ (MAPI).

Figure 3 shows the absorption front of the 800 nm thick MAPI layer and its photoluminescence (PL) spectrum at room temperature just after the deposition at time t = 0 day. The layer presents a PL spectrum centered at 1.64 eV. The absorption and PL spectra are quite coherent with the spectra commonly seen in the literature [21].

**Figure 4:**
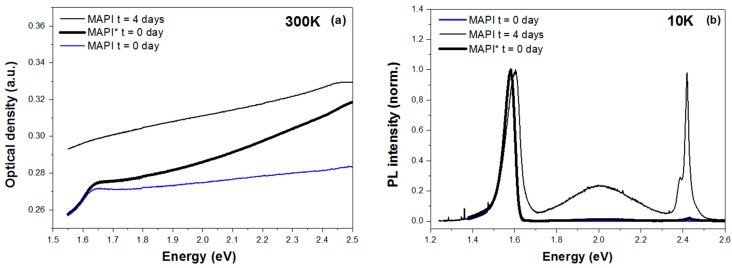

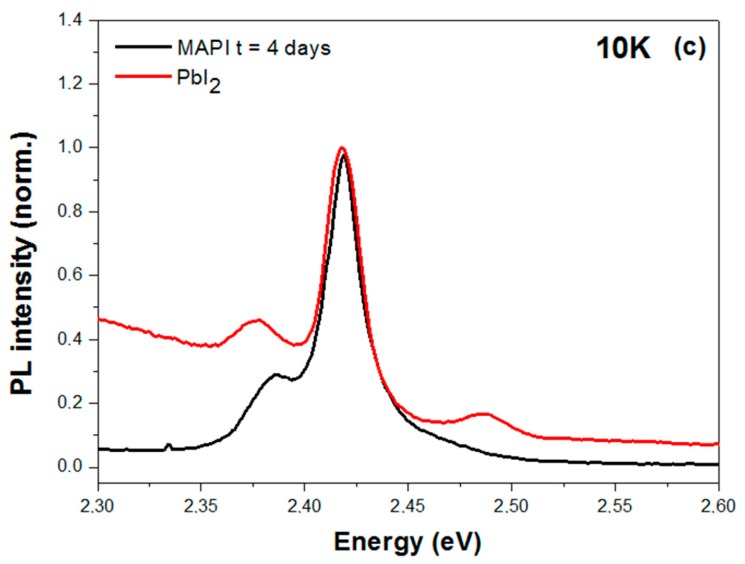
Absorption at room temperature (**a**); and (**b**) PL spectra at low temperature 10 K (excitation at 3.815 eV for the PL) of a 800 nm thick MAPI layer, at t = 0 day (blue line) and t = 4 days (black line). The bold line shows the spectra for MAPI* sample obtained with the powdered PbI_2_. The PL spectra have been normalized so that the maximum PL at 1.64 eV is 1; (**c**) PL spectra at 10 K in the 2.3–2.6 eV range of MAPI layer at t = 4 days and PbI_2_. The PL spectra have been normalized so that the maximum PL at 2.4 eV is 1.

Figure 4a shows the evolution of the absorption front of the MAPI thin layer at room temperature after 4 days: the absorption front disappears when the perovskite degrades. After 4 days, a feature around 2.5 eV appears in the absorption spectrum, corresponding to the presence of PbI_2_. In a correlated way, two new features appear in the PL spectrum at low temperature shown in Figure 4b: a broad PL signal between 1.9 eV and 2.2 eV and a narrow PL peaks around 2.4 eV. These features are quite similar to the PL spectra obtained at 15 K of 60 µm PbI_2_ layers produced by the spray pyrolysis technique [41]. We have deposited a 200 nm thick PbI_2_ layer by spin-coating on a quartz substrate, and registered its PL spectrum at 10 K (excitation wavelength at 325 nm, HeCd laser). Figure 4c shows the PL spectrum of the PbI_2_ layer in the 2.3–2.6 eV range, superimposed to the MAPI PL spectrum at t = 4 days. Clearly, the PL peak at 2.4 eV is characteristic of the PbI_2_ luminescence and can be taken as the signature of PbI_2_ presence.

**Figure 5:**
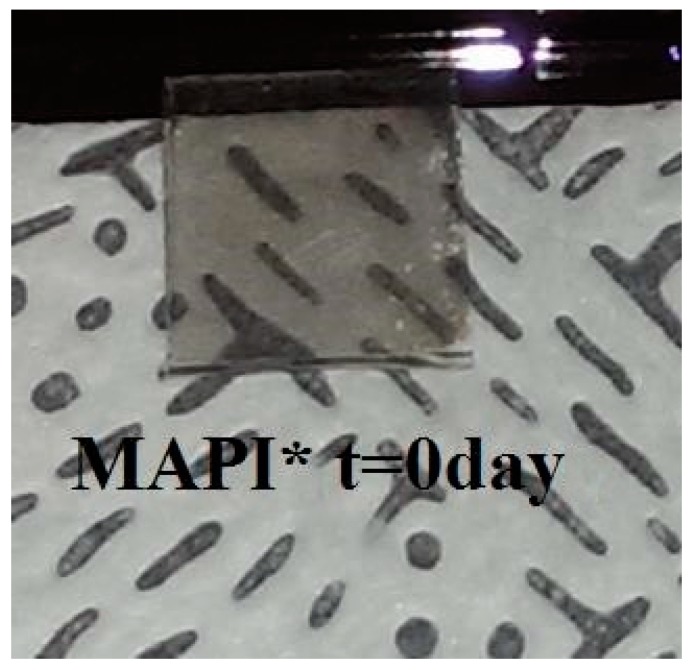
Photograph of the MAPI* thin layer at t = 0 day.

This experimental method of detection of PbI_2_ traces could be also used to determine rapidly and easily if some PbI_2_ clusters remain after a two-step deposition method, such as the one developed by Wu et al. [35], for example. It can also outline the influence of the precursors: for example, here we study the influence of the texture of the PbI_2_ which is used to prepare the CH_3_NH_3_PbI_3_ layers. Let us compare the MAPI sample prepared PbI_2_, in forms of grains, and the MAPI* sample prepared with powdered PbI_2_ (same deposition method with the same parameters and same thickness for the two samples). Figure 4b shows the PL spectrum of these two samples just after the deposition (t = 0 day). It can be seen that we can detect PbI_2_ traces in MAPI* sample and no PbI_2_ traces in the MAPI sample, showing that the choice of the PbI_2_ origin and conditioning is important to obtain PbI_2_-free samples at t = 0 day. In the following samples presented in this paper, we will use grains of PbI_2_ for the preparation. Moreover, observing the Figure 5 exhibiting the photography of the MAPI* sample, it has to be pointed out that there is no evidence, looking with naked eyes, of the presence of PbI_2_ traces in the sample.

**Figure 6:**
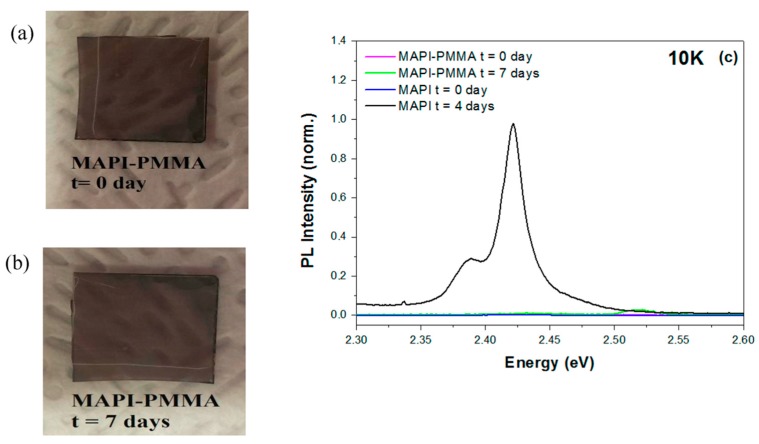
Photographs at (**a**) t = 0 day; (**b**) t = 7 days of a 800 nm thick MAPI layer overcoated with a 200 nm thick PMMA layer (called MAPI-PMMA); (**c**) PL spectra (excitation at 3.815 eV) in the 2.3–2.6 eV range for MAPI-PMMA at t = 0 day and MAPI-PMMA at t = 7 days, MAPI t = 4 days is recalled here. The PL spectra have been normalized so that the maximum PL at 1.64 eV is 1 (I_1.64 eV_ = 1).

In order to apply this method of using the PL spectroscopy at low temperature to detect traces of PbI_2_, we also studied the aging of MAPI thin layers, protected from atmosphere moisture or ambient light, as a great number of papers consider the important role of these two parameters. Firstly, we protect the MAPI layer with a 200 nm thick polymethylmetacrylate (PMMA) layer deposited by spin-coating over the 800 nm thick MAPI layer: the deposition parameters of the 4% wt PMMA solution in toluene are 1000 rpm for 40 s, annealed at 95 °C for 20 min. The PMMA protected sample will be called MAPI-PMMA. Figure 6a,b show the photographs of the MAPI-PMMA layer at t = 0 day and t = 7 days respectively: the sample doesn’t appear yellow, even after 7 days. To check if there are some PbI_2_ traces, we have performed the PL spectrum at low temperature: in Figure 6c, no PbI_2_ PL signal can be detected, so that we can affirm that no PbI_2_ formation has occurred in this sample after 7 days. We have done the experiment up to 30 days and no PbI_2_ traces have been detected. As a consequence, we can conclude the coating of the perovskite with a 200 nm PMMA layer is efficient to protect from the degradation of the sample due to the atmospheric moisture. We have also protected an identical MAPI layer with a 300 nm thick Spiro-OMeTAD layer, which is a Hole Transport Layer commonly used in hybrid perovskite solar cells. After 17 days of aging in ambient atmosphere, we can conclude that no PbI_2_ luminescence has been detected.

**Figure 7:**
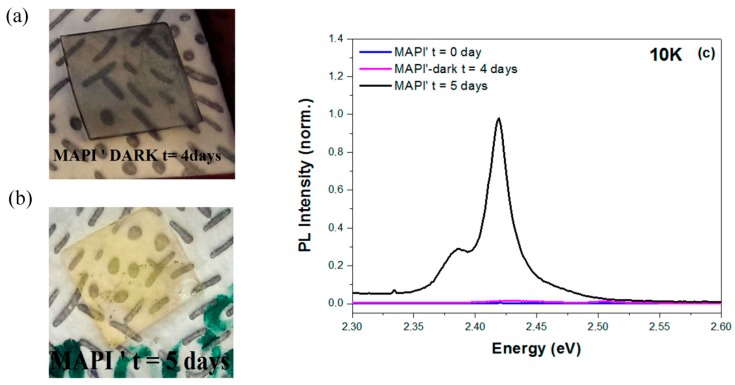
Photographs of (**a**) a 800 nm thick MAPI layer stored in a dark box at t = 4 days; (**b**) a 800 thick MAPI layer stored in a transparent box, at t = 5 days; (**c**) PL spectra (excitation at 3.815 eV) in the 2.3–2.6 eV range for MAPI layer in the dark box at t = 4 days (MAPI’-dark), MAPI layer in the transparent box (MAPI’) at t = 5 days. The PL spectra have been normalized so that the maximum PL at 1.64 eV is 1 (I_1.64 eV_ = 1).

Secondly, we tried to evaluate the role of light exposition. For this, we studied the degradation of unprotected samples in the ambient light, or in the dark: we thus prepared a 800 nm thick MAPI layer on a quartz substrate, and cut the sample in two pieces, respectively stored in a dark box (MAPI’-dark), and in a transparent box (MAPI’). It can be seen in Figure 7 that after 4 days, the sample stored in the black box has not aged (no PbI_2_ detected), at the difference of the sample stored in the transparent box.

**Figure 8:**
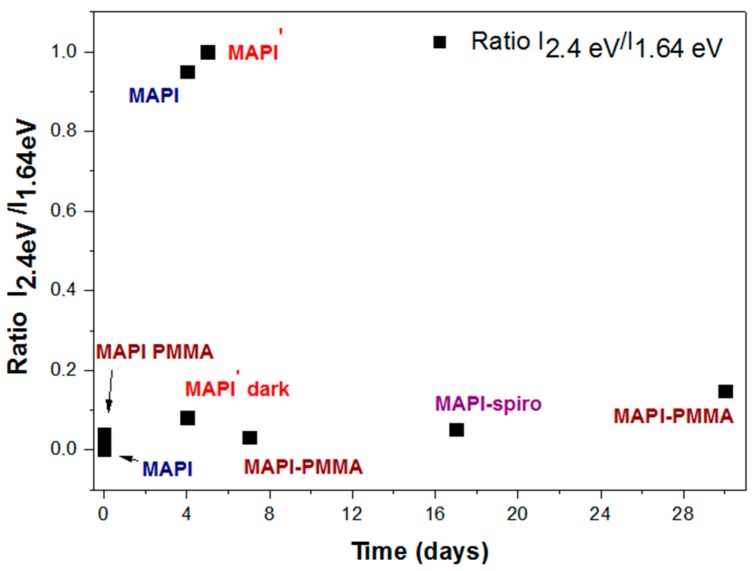
Values of the ratio (I_2.4 eV_/I_1.64 eV_) for each considered samples.

As the above PL spectra in Figure 6c and Figure 7c have been normalized (I_1.64 eV_ = 1), the ratio of the PL intensity at 2.4 eV and the PL intensity at 1.64 eV: I_2.4 eV_/I_1.64 eV_, is directly related to the quantity of the degraded hybrid perovskite. Figure 8 shows the values of this ratio for each considered sample. On this kind of diagram, one can read easily the evolution of the degradation of the MAPI layers over time; when the degradation level is small, the PbI_2_ amount generated is also small and very divided, and therefore the reabsorption (by PbI_2_) can be neglected and the measurements can be considered as sincere; at high degradation rates this is probably not accurate any longer, and some intrinsic error might exist, however the result still display a good approximation. We read synthetically that PMMA and Spiro-OMeTAD layers are able to protect the MAPI layer and that the mechanism which is involved in the degradation of MAPI and the appearance of PbI_2_ is activated by light.

### 2.2. Optical Properties and Stability of Millimeter Sized Crystals

**Figure 9:**
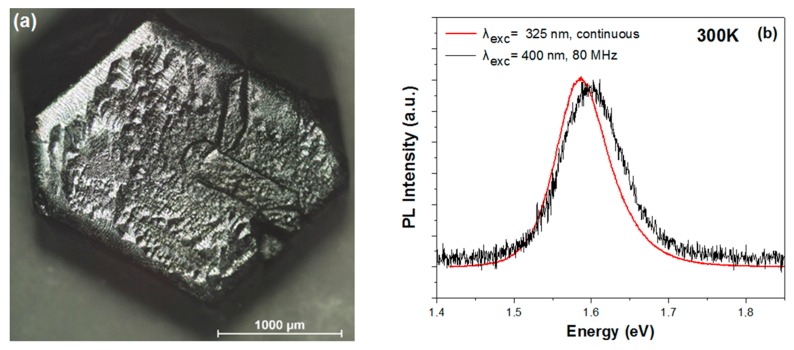
(**a**) Microscopy image of a millimeter sized MAPI crystal; (**b**) PL spectrum at room temperature of this MAPI crystal, taken six months after its growth, under continuous excitation at E_exc_ = 3.815 eV (λ_exc_ = 325 nm, HeCd laser) (red line) and quasi-continuous excitation at E_exc_ = 3.1 eV (λ_exc_ = 400 nm) with a 80 MHz doubled-frequency pulsed Ti-Sa laser (black line).

**Figure 10:**
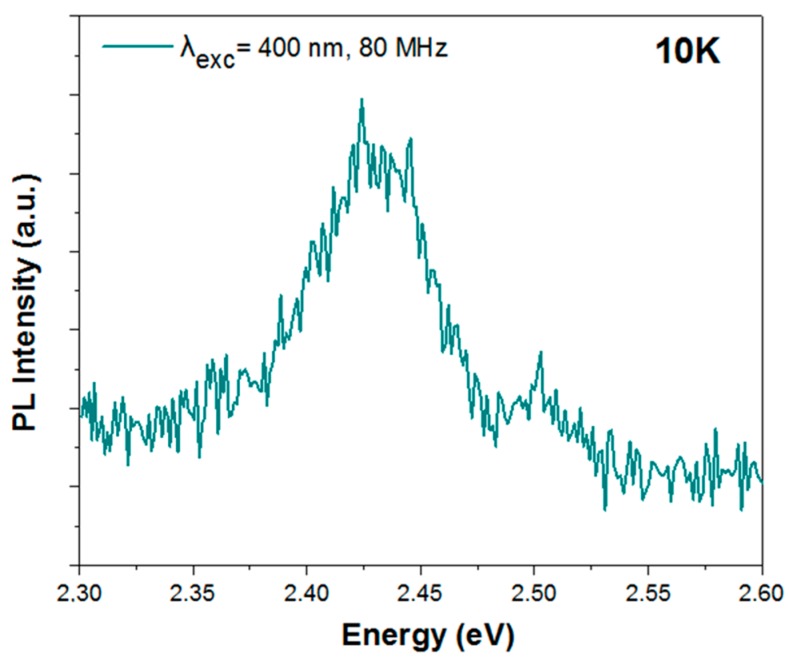
PL spectrum of the millimeter sized MAPI crystal at 10 K, taken six months after its growth under quasi-continuous excitation at 3.1 eV (400 nm) with a 80 MHz doubled-frequency pulsed Ti-Sa laser.

Figure 9a shows the optical microscopy image of a millimeter sized MAPI crystal, taken 6 months after its growth. No precautions were taken for the storage of this sample during these 6 months: the sample has been stored in a transparent box, and left under ambient conditions. Although the growth of this crystal has been done in solution for a long time, and despite the absence of precautions for the storage, no PbI_2_ traces are detectable by naked eye. Figure 9b shows the PL spectrum at room temperature of the millimeter sized crystal shown in Figure 9a, under different excitation conditions: a continuous excitation at 3.815 eV (325 nm) obtained with a HeCd laser and a quasi-continuous excitation at 3.1 eV (400 nm) with a doubled-frequency pulsed Ti-Sa laser. For both considered excitation conditions, the emission of the crystal was recorded centered at the same wavelength than the one of the previously studied MAPI thin layers. In order to check if PbI_2_ traces can be detected in this monocrystal, the PL spectrum has been done at low temperature: in Figure 10, some extremely small traces of PbI_2_ can be detected. In a crystal, it is difficult to extract quantitative data because some reabsorption of the PbI_2_ luminescence can occur, but at least, we can affirm that small PbI_2_ traces are present in the sample.

Such small PbI_2_ traces could not be observed by X-diffraction. These traces couldn’t be either detected from the absorption spectrum. Indeed, from Figure 3 and Figure 4a, the order of magnitude of the MAPI absorption coefficient is evaluated in the 2.4 eV range: 3750 cm^−1^, that is to say that the optical density for a crystal having a thickness of 2 mm will be 750, making impossible to detect weak signals coming from PbI_2_ absorption. The low temperature PL is therefore here the only mean to detect these weak PbI_2_ traces.

PL spectroscopy is a powerful tool to detect weak signals because it is a signal detection method requiring a black background: in the range of wavelengths where the sample shows no emission, the emission signal is strictly zero, as a consequence, if a small emission signal exists for certain wavelengths, it will be possible to increase the detector sensitivity in order to detect the emission signal. It is quite impossible to do that in an absorption experiment: in the range of wavelengths where the sample shows no absorption, the incident light is completely transmitted, so a high signal arrives in the detector. If a small absorption exists at a certain wavelength, the incident light will be nearly completely transmitted, as a consequence it will be impossible to play with the detector sensitivity to detect the very weak absorption signal among the large transmitted signal. It is the reason why PL spectroscopy is much more sensitive than absorption spectroscopy to detect weak signals in solids. Additionally, the low temperature PL spectroscopy can be performed in situ, in the cryostat. In fact, in order to optimize the solar cells performances, advanced optical studies are necessary: for example, it is crucial to understand the nature and the dynamics of the relaxation mechanisms involved in the trapping of excitons on defects, it is also important to study some behaviors of the free excitons, such as their diffusion length. To perform these studies, time-resolved PL experiments at low temperature are necessary [42,43]. In this case, the method we propose here allows to check, in a simple way and in situ, the presence of PbI_2_ traces.

## 3. Discussion on MAPI Degradation Mechanisms

**Scheme 1:**
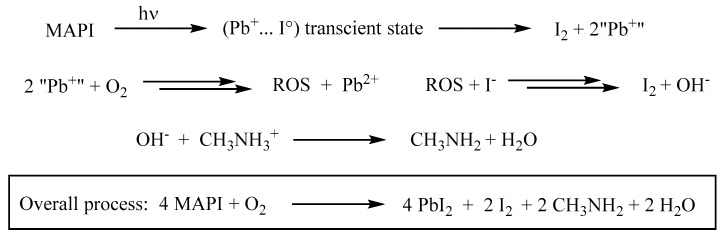
Degradation processes susceptible to occur upon the photochemical degradation of MAPI, either involving simply water (top Scheme) or oxygen (bottom Scheme). ROS stands for Reactive Oxygen Species.

**Scheme 2:**
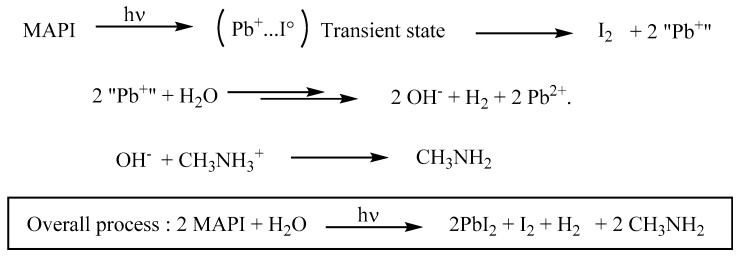
Alternative degradation mechanism of MAPI with production of hydrogen.

The experiments performed on MAPI thin layers showed that PMMA and Spiro-OMeTAD layers are both able to protect the MAPI layer and that the mechanism which is involved in the degradation of MAPI and the appearance of PbI_2_ is activated by light. As we have demonstrated in the case of 2D hybrid perovskite [44], the primary process in perovskite degradation is the (minor) evolution of the photogenerated charge transfer excited state “Pb^+^…I°” in the material, generating I_2_ which goes off. The fate of the transient very unstable “Pb^+^” species is not clear, but it is likely that it reacts in air with the strongest easily diffusing oxidizing reagent, that is oxygen. ROS species (O_2_^−^, HO_2_^−^…) can then be formed, which in turn oxidize iodides, and in the end generate hydroxide ions. These hydroxide ions deprotonate the ammoniums (likely a slower process in the solid matrix), and the low boiling methylamine also goes off. In the end, only PbI_2_ is left. In equations, the picture can be summarized as (overall stoichiometry only in the circled global Scheme 1 and Scheme 2):

This mechanism is consistent with the work of Haque and co-workers who argued that under illumination, photoexcited electrons would react with molecular oxygen to form superoxide (O_2_^−^) that would subsequently decompose the perovskite [45]. On the other hand, Christians et al. [37] have convincingly demonstrated that formation of a perovskite hydrate in moist air occurred, and that this was linked to the fast photodegradation of the material afterwards. In that case, the water present in the structure might well be the reducing agent, which would trigger an alternative mechanism, a little slower and producing dihydrogen instead of water.

Lastly, it should be noticed that the PMMA covered MAPI layer is slowly photodegraded, since PMMA completely protects from moisture, but not from oxygen; in addition, nobody has never reported the production of hydrogen gas, which tends to suggest that the second mechanism would be a minor one. Finally, the two mechanisms can exist concomitantly and present synergy. Even if water is not the active agent in the degradation process, it might well help the diffusion of both oxygen and hydroxide ions to accelerate the overall process.

**Figure 11:**
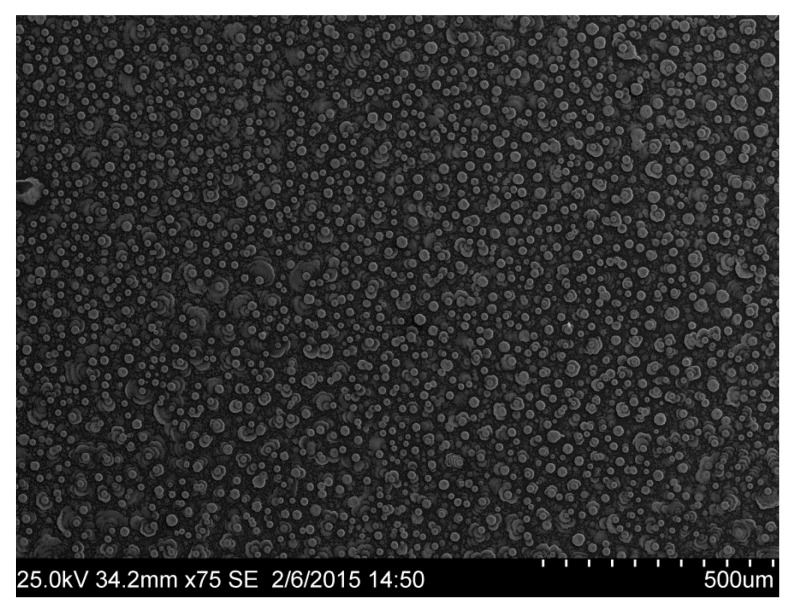
Scanning Electron Microscopy (SEM) image of an 800 thick MAPI thin layer deposited by spin-coating on a quartz substrate.

From the experiments on MAPI crystals, the highlight is the extremely high stability of the crystals compared to the thin layers. In a crystal, an intimate contact with water is much more difficult than in a thin layer (the insertion of water would probably provoke a crystal breakdown). On the other hand, Figure 11 shows a Scanning Electron Microscopy (SEM) image of a MAPI thin layer, showing the large surface that can be in contact with water and above all the great number of grain boundaries.

As our millimeter crystals are single crystals presenting a low density of defects and likely have no grain boundaries, we conclude that these defects and grain boundaries play an important role in the degradation mechanism and that growing very high quality perovskite films in solar cells is therefore necessary to avoid degradation. This finding is quite coherent with recent interpretations. In their studies about degradation mechanisms of MAPI in thin layers and crystals, Leguy et al. [38] suggested a high degree of penetration of water molecules into MAPI thin films, arising probably from the diffusion of water molecules along the grain boundaries. Lu et al. [39] have studied MAPI thin layers deposited by two different methods, one of this method producing higher quality thin layers: they observe a more important interaction with moisture in the layers containing more defects, providing in their opinion more active sites for the adsorption of water molecules.

## 4. Materials and Methods

### 4.1. Preparation of Perovskite Precursors

First of all, methylammonium iodide CH_3_NH_3_I (called MAI hereafter) is synthetized by reacting CH_3_NH_2_ (30 mL, 2 M in methanol, Sigma Aldrich, St. Louis, MO, USA) and aqueous solution of iodic acid (HI, 34.4 mL 57% in water, Sigma Aldrich) in a 250 mL round-bottom flask, stirred at 0 °C for 4 h. The precipitate is recovered after evaporation at 60 °C for 1 h. Then the MAI is dissolved in ethanol and recrystallized three times from diethyl ether. The final product is dried at 60 °C in vacuum oven for 24 h. To prepare the perovskite CH_3_NH_3_ PbI_3_ (MAPI), the synthesized CH_3_NH_3_I (0.5 g) and lead iodide (PbI_2_, 1.47 g of grains from Sigma Aldrich 99.99% for all the samples except for the sample called MAPI*, for which powdered Sigma-Aldrich 99.99% PbI_2_ was used) are mixed in γ-butyrolactone (4.47 mL, Sigma Aldrich reagent plus > 99%) (stoichiometric amounts) and stirred at 60 °C for 12 h. The obtained solution is homogeneous with high transparency in the visible region, and remains totally clear when it is stored during a long time (more than one month), that is to say that no PbI_2_ is formed inside the solution.

### 4.2. Deposition of Perovskite Layer

UV quartz slides (from Neyco, Vanves, France) are used for deposition. They are cleaned sequentially with acetone, ethanol and propanol in an ultrasonic bath (15 min for each step). Finally the slides are immerded in 1 M KOH in ethanol for 15 min. Then the slides are rinsed with distilled water and dried by a nitrogen gas flow. MAPI films are deposited on these quartz substrates by spin-coating, adjusting the acceleration and duration of the acceleration to obtain the desired thickness. After the deposition, the samples are annealed at 120 °C for 10 min. We focus the paper on layers of thickness 800 nm as the thickness of the MAPI layers in the solar cells is typically several hundred of nm. These layers are obtained with spin-coater parameters of 2000 rpm for 10 s, the thickness has been checked by AFM measurements.

### 4.3. Preparation of Millimeter Sized Crystals

On the basis of articles recently published [46,47,48,49], we have grown millimeter sized MAPI crystals. Methylammonium iodide (0.78 g, 5 mmol) and lead iodide (2.30 g, 5 mmol) were dissolved in GBL (5 mL) at 60 °C. The yellow solution (2 mL) was placed in a vial and heated at 120 °C during one to four hours depending on the desired crystal size. The solution could be heated in a hot plate as well as an oil bath.

## 5. Conclusions

Investigating the stability and evaluating the quality of the perovskite structures is quite critical both for the design and fabrication of high-performance perovskite devices and for fundamental studies of the photophysics of the excitons. We have shown that low temperature PL spectroscopy is a powerful tool to detect PbI_2_ traces in hybrid perovskite layers and crystals. Because PL spectroscopy is a signal detection method on a black background, small PbI_2_ traces can be detected, when other methods, currently used at room temperature, fail. Additionally, in the context of advanced optical studies of the excitons, PL spectroscopy allows to check in situ the presence of PbI_2_ in samples in a very simple way. In particular, this method is quite convenient for the study of the crystal aging and quality, as X-diffraction and absorption spectroscopies are inadequate to detect the presence of PbI_2_ traces in this case.

This method has been applied to study MAPI thin layers and single crystals. The processes involved in the degradation likely involve dioxygen, or water, but more likely the two together, with mutual enhancement, the water helping oxygen, the strongest oxidant, to reach reactive zones localized on defects. Our study highlights the extremely high stability of the single crystals compared to the thin layers. So defects and grain boundaries are thought to play an important role in the degradation mechanism. As a consequence, growing very high quality perovskite films in solar cells is quite necessary to avoid degradation and increase the resistance to moisture.
